# 2,2′-(3,5-Dinitro­benzyl­imino)diethanol

**DOI:** 10.1107/S1600536808017066

**Published:** 2008-06-13

**Authors:** Gul S. Khan, George R. Clark, David Barker

**Affiliations:** a Chemistry Department, The University of Auckland, Private Bag 92019, Auckland, New Zealand

## Abstract

The title compound, C_11_H_15_N_3_O_6_, was prepared by the reaction of (3,5-dinitro­benz­yl)methane­sulfonate with diethanol­amine. The asymmetric unit contains four crystallographically independent mol­ecules which differ primarily in their rotation about the bond between the aromatic ring and the *N*-diethanol unit. The mol­ecules are linked into sheets by a hydrogen-bonding network which involves all of the hydroxy groups, with only van der Waals contacts between the sheets.

## Related literature

For the structure of a mononitro derivative, see: Blake *et al.* (1998 ▶). For the incorporation of *N*,*N*-bis­(2-hydroxy­ethyl)benzyl­amines in macromolecular metal complexes, see: Koizumi *et al.* (2005 ▶, 2007 ▶). For the use of *N*,*N*-bis­(2-hydroxy­ethyl)nitro­benzyl­amines as nitro­gen mustard precursors, see: Bacherikov *et al.* (2005 ▶); Garg *et al.* (1976 ▶); Tercel *et al.* (1996 ▶); Wilson & Tishler (1951 ▶). For related literature, see: Crans & Boukhobza (1998 ▶); Kagitani *et al.* (1986 ▶).
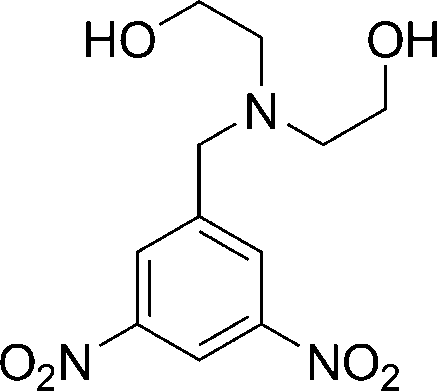


## Experimental

### 

#### Crystal data

C_11_H_15_N_3_O_6_
*M* *_r_* = 285.26
Triclinic, 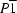

*a* = 12.8042 (3) Å
*b* = 14.7498 (3) Å
*c* = 15.1282 (4) Å
α = 104.141 (1)°
β = 96.371 (1)°
γ = 106.334 (1)°
*V* = 2608.67 (11) Å^3^
*Z* = 8
Mo *K*α radiation
μ = 0.12 mm^−1^
*T* = 84 (1) K
0.30 × 0.30 × 0.24 mm

#### Data collection

Bruker SMART CCD diffractometer
Absorption correction: multi-scan (*SADABS*; Sheldrick, 1997 ▶) *T*_min_ = 0.795, *T*_max_ = 0.977
24719 measured reflections
10576 independent reflections
8548 reflections with *I* > 2σ(*I*)
*R*_int_ = 0.024

#### Refinement

*R*[*F* ^2^ > 2σ(*F* ^2^)] = 0.046
*wR*(*F* ^2^) = 0.101
*S* = 1.08
10576 reflections
753 parameters
H atoms treated by a mixture of independent and constrained refinement
Δρ_max_ = 0.33 e Å^−3^
Δρ_min_ = −0.25 e Å^−3^

### 

Data collection: *SMART* (Bruker, 1995 ▶); cell refinement: *SAINT* (Bruker, 1995 ▶); data reduction: *SAINT*; program(s) used to solve structure: *SHELXS97* (Sheldrick, 2008 ▶); program(s) used to refine structure: *SHELXL97* (Sheldrick, 2008 ▶); molecular graphics: *ORTEPIII* (Burnett & Johnson, 1996 ▶); software used to prepare material for publication: *SHELXTL* (Sheldrick, 2008 ▶).

## Supplementary Material

Crystal structure: contains datablocks I, global. DOI: 10.1107/S1600536808017066/hg2406sup1.cif

Structure factors: contains datablocks I. DOI: 10.1107/S1600536808017066/hg2406Isup2.hkl

Additional supplementary materials:  crystallographic information; 3D view; checkCIF report

## Figures and Tables

**Table 1:** Hydrogen-bond geometry (Å, °)

*D*—H⋯*A*	*D*—H	H⋯*A*	*D*⋯*A*	*D*—H⋯*A*
O5*A*—H*O*5*A*⋯O6*C*^i^	0.89 (3)	1.85 (3)	2.7343 (19)	174 (2)
O6*A*—H*O*6*A*⋯O5*D*	0.84 (3)	1.93 (3)	2.7575 (19)	174 (3)
O5*B*—H*O*5*B*⋯O5*A*^ii^	0.84 (3)	2.07 (3)	2.8892 (19)	165 (2)
O6*B*—H*O*6*B*⋯O6*D*	0.87 (3)	1.89 (3)	2.7529 (19)	170 (3)
O5*C*—H*O*5*C*⋯O5*A*^i^	0.85 (3)	2.02 (3)	2.8643 (19)	171 (2)
O6*C*—H*O*6*C*⋯O6*A*^i^	0.87 (3)	1.89 (3)	2.7553 (19)	170 (2)
O5*D*—H*O*5*D*⋯O6*B*	0.87 (3)	1.94 (3)	2.802 (2)	170 (2)
O6*D*—H*O*6*D*⋯O5*B*	0.88 (3)	1.90 (3)	2.7804 (19)	173 (3)

Symmetry codes: (i) 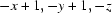; (ii) 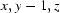.

**Fig. 1.**
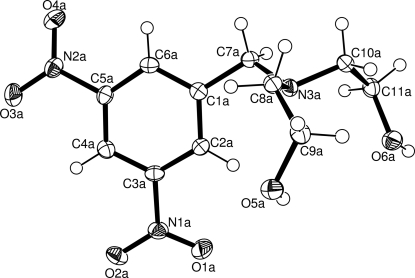
: Structure showing 50% probability displacement ellipsoids for non-hydrogen atoms and hydrogen atoms as arbitary spheres for Molecule A (Burnett & Johnson, 1996).

## supplementary crystallographic information

### Comment

N,N-Bis(2-hydroxyethyl)amines are commonly used precusors for the synthesis of N,N-bis(2-chloroethyl)amines, more commonly known as nitrogen mustards. In most cases the amine is an aniline however there are cases where the more reactive benzyl amines have been used (Bacherikov et al.,  2005, Garg et al., 1976, Tercel et al., 1996, Wilson & Tishler, 1951). The use of dinitrobenzylamines, including an isomer of the title comound, N,N-bis(2-hydroxyethyl)-3,4-dinitrobenzylamine, as radiosensitizers has been reported (Kagitani et al. 1986). The dual functionality of the two free hydroxyl groups along with a basic nitrogen have seen N,N-bis(2-hydroxyethyl) benzylamines used in synthesis of numerous metal complexes including those containing vandium (Crans & Boukhobza, 1998), manganese (Koizumi et al., 2005, 2007) and iron (Koizumi et al., 2005).

The crystals contain four crystallographically independent molecules which differ primarily in their rotation about the bond between the aromatic ring and the N-diol moiety. The molecules are linked into sheets by a hydrogen bonding network which involves all of the diols, with only van der Waals contacts between the sheets. The four molecules differ in their rotation about the C1-C7 bond (torsion angles C2-C1-C7-N3 for molecules A,B,C, and D are -38, 53, 47, and -59 degrees respectively). The X-ray crystal structure of a mono-nitro derivative has been reported previously (Blake et al., 1998).

### Experimental

To a solution of diethanolamine (2.69 g, 25.34 mmol) in dry THF (10 ml), at 273K, under an atmosphere of nitrogen, was added dropwise a solution of 3,5-dinitrobenzyl methanesulfonate (700 mg, 2.53 mmol) in dry THF (5 ml), and the mixture stirred at room temperature for 24 hr. The solvent was removed *in vacuo*, the residue diluted with ethyl acetate (30 ml) and 2*M* hydrochloric acid (15 ml), and the aqueous layer separated. The aqueous extract was neutralized with 4 *M* sodium hydroxide and extracted with ethyl acetate (3 *x* 40 ml). The combined organic extracts were dried (Na_2_SO_4_) and the solvent removed *in vacuo* to afford the crude product, which was purified by flash chromatography (9:1 dichloromethane-methanol) to afford the title compound (710 mg, 98%) as a yellow solid) which was recrystallized from dichloromethane/chloroform to give light yellow crystals (m.p. 350–351 K) suitable for X-ray crystallography. IR ν_max_ (NaCl)/cm^-1^ 3427, 2955, 1645, 1535. ^1^H NMR (400 MHz, CDCl_3_, δ, p.p.m.) 2.73 (4*H*, t, J = 10.1 Hz, N(C*H*_2_CH_2_OH)_2_), 3.67 (4*H*, t, J = 10.1 Hz, N(CH_2_C*H*_2_OH)_2_), 3.92 (2*H*, s, ArCH_2_N), 8.61 (2*H*, br s, Ar—H), 8.88 (1*H*, br s, Ar—H; ^13^C NMR (400 MHz, CDCl_3_, δ, p.p.m.) 55.9 (CH_2_, N(*C*H_2_CH_2_OH)_2_), 58.4 (CH_2_, Ar*C*H_2_N), 59.6 (CH_2_, N(CH_2_*C*H_2_OH)_2_), 117.6 (CH, Ar—C), 128.7 (CH, Ar—C), 144.6 (quat., Ar—C), 148.5 (quat., Ar—C). MS m/*z* (FAB) 286 (MH^+^, 20%), 254 (M—CH_2_OH, 10), 154 (100). HRMS (FAB) Found MH^+^ 286.10436. C_11_H_16_N_3_O_6_ requires 286.10391.

### Refinement

Geometrically constrained hydrogen atoms were placed in calculated positions and refined using the riding model [C—H 0.93–0.97 Å), with *U*_iso_(H) = 1.2 or 1.5 times *U*_eq_(C). The hydrogen atoms of the diol moieties were located in a difference Fourier map and refined individually with isotropic temperature factors.

### Crystal data

**Table:**

C_11_H_15_N_3_O_6_	*Z* = 8
*M**_r_* = 285.26	*F*_000_ = 1200
Triclinic, *P*1	*D*_x_ = 1.453 Mg m^−^^3^
Hall symbol: -P 1	Mo *K*α radiation λ = 0.71073 Å
*a* = 12.8042 (3) Å	Cell parameters from 7193 reflections
*b* = 14.7498 (3) Å	θ = 1.5–26.4º
*c* = 15.1282 (4) Å	µ = 0.12 mm^−^^1^
α = 104.141 (1)º	*T* = 84 (1) K
β = 96.371 (1)º	Block, yellow
γ = 106.334 (1)º	0.30 × 0.30 × 0.24 mm
*V* = 2608.67 (11) Å^3^	

### Data collection

**Table:**

Bruker SMART CCD diffractometer	10576 independent reflections
Radiation source: fine-focus sealed tube	8548 reflections with *I* > 2σ(*I*)
Monochromator: graphite	*R*_int_ = 0.024
*T* = 84(1) K	θ_max_ = 26.4º
Area–detector ω scans	θ_min_ = 1.5º
Absorption correction: multi-scan(SADABS; Sheldrick, 1997)	*h* = −16→16
*T*_min_ = 0.795, *T*_max_ = 0.977	*k* = −18→17
24719 measured reflections	*l* = −18→18

### Refinement

**Table:**

Refinement on *F*^2^	Secondary atom site location: difference Fourier map
Least-squares matrix: full	Hydrogen site location: inferred from neighbouring sites
*R*[*F*^2^ > 2σ(*F*^2^)] = 0.047	H atoms treated by a mixture of independent and constrained refinement
*wR*(*F*^2^) = 0.101	*w* = 1/[σ^2^(*F*_o_^2^) + (0.0308*P*)^2^ + 1.4297*P*] where *P* = (*F*_o_^2^ + 2*F*_c_^2^)/3
*S* = 1.08	(Δ/σ)_max_ < 0.001
10576 reflections	Δρ_max_ = 0.33 e Å^−^^3^
753 parameters	Δρ_min_ = −0.25 e Å^−^^3^
Primary atom site location: structure-invariant direct methods	Extinction correction: none

### Special details

**Table:**

Geometry. All e.s.d.'s (except the e.s.d. in the dihedral angle between two l.s. planes) are estimated using the full covariance matrix. The cell e.s.d.'s are taken into account individually in the estimation of e.s.d.'s in distances, angles and torsion angles; correlations between e.s.d.'s in cell parameters are only used when they are defined by crystal symmetry. An approximate (isotropic) treatment of cell e.s.d.'s is used for estimating e.s.d.'s involving l.s. planes.
Refinement. Refinement of *F*^2^ against ALL reflections. The weighted *R*-factor *wR* and goodness of fit *S* are based on *F*^2^, conventional *R*-factors *R* are based on *F*, with *F* set to zero for negative *F*^2^. The threshold expression of *F*^2^ > σ(*F*^2^) is used only for calculating *R*-factors(gt) *etc*. and is not relevant to the choice of reflections for refinement. *R*-factors based on *F*^2^ are statistically about twice as large as those based on *F*, and *R*- factors based on ALL data will be even larger.

### Fractional atomic coordinates and isotropic or equivalent isotropic displacement parameters (Å^2^)

**Table:**

	*x*	*y*	*z*	*U*_iso_*/*U*_eq_	
O1A	0.22043 (12)	0.88717 (13)	0.52205 (10)	0.0424 (4)	
O2A	0.13995 (11)	0.97382 (10)	0.61100 (9)	0.0278 (3)	
O3A	−0.25699 (12)	0.90173 (11)	0.52437 (9)	0.0331 (3)	
O4A	−0.34809 (11)	0.78156 (11)	0.40131 (10)	0.0326 (3)	
O5A	0.20448 (11)	0.95928 (10)	0.24284 (9)	0.0238 (3)	
HO5A	0.228 (2)	0.9201 (19)	0.2705 (17)	0.049 (7)*	
O6A	0.23610 (11)	0.66199 (10)	0.17337 (9)	0.0255 (3)	
HO6A	0.271 (2)	0.626 (2)	0.1880 (18)	0.053 (8)*	
N1A	0.13972 (13)	0.91221 (12)	0.53943 (11)	0.0244 (3)	
N2A	−0.26153 (13)	0.83747 (12)	0.45355 (11)	0.0240 (3)	
N3A	0.04351 (12)	0.74465 (11)	0.18600 (10)	0.0178 (3)	
C1A	−0.05692 (15)	0.76440 (13)	0.31650 (12)	0.0183 (4)	
C2A	0.04103 (15)	0.81190 (13)	0.38236 (12)	0.0184 (4)	
H2A	0.1086	0.8082	0.3675	0.022*	
C3A	0.03615 (15)	0.86465 (13)	0.47012 (12)	0.0193 (4)	
C4A	−0.06087 (15)	0.87488 (13)	0.49663 (12)	0.0208 (4)	
H4A	−0.0619	0.9117	0.5558	0.025*	
C5A	−0.15596 (15)	0.82691 (13)	0.42957 (12)	0.0200 (4)	
C6A	−0.15707 (15)	0.77181 (13)	0.34075 (12)	0.0196 (4)	
H6A	−0.2234	0.7403	0.2980	0.023*	
C7A	−0.05503 (15)	0.70189 (13)	0.22151 (12)	0.0198 (4)	
H7A1	−0.0570	0.6366	0.2250	0.024*	
H7A2	−0.1209	0.6947	0.1784	0.024*	
C8A	0.03299 (15)	0.82865 (13)	0.15390 (12)	0.0200 (4)	
H8A1	−0.0046	0.8645	0.1947	0.024*	
H8A2	−0.0124	0.8040	0.0920	0.024*	
C9A	0.14419 (15)	0.89869 (13)	0.15193 (12)	0.0222 (4)	
H9A1	0.1884	0.8607	0.1228	0.027*	
H9A2	0.1325	0.9409	0.1142	0.027*	
C10A	0.05911 (15)	0.66762 (13)	0.11017 (12)	0.0197 (4)	
H10A	0.1003	0.6988	0.0695	0.024*	
H10B	−0.0129	0.6249	0.0738	0.024*	
C11A	0.12023 (15)	0.60617 (13)	0.14604 (13)	0.0218 (4)	
H11A	0.0919	0.5893	0.1988	0.026*	
H11B	0.1094	0.5454	0.0979	0.026*	
O1B	0.25171 (11)	0.47448 (12)	0.53893 (10)	0.0359 (4)	
O2B	0.17606 (12)	0.46382 (11)	0.65871 (9)	0.0324 (3)	
O3B	−0.21833 (12)	0.36844 (12)	0.62284 (9)	0.0367 (4)	
O4B	−0.32339 (11)	0.30873 (11)	0.48596 (10)	0.0318 (3)	
O5B	0.10813 (11)	0.11492 (10)	0.29582 (9)	0.0231 (3)	
HO5B	0.146 (2)	0.0764 (19)	0.2885 (17)	0.045 (7)*	
O6B	0.21700 (11)	0.35414 (10)	0.16911 (9)	0.0263 (3)	
HO6B	0.216 (2)	0.339 (2)	0.2213 (19)	0.057 (8)*	
N1B	0.17109 (13)	0.45026 (11)	0.57475 (11)	0.0235 (3)	
N2B	−0.23257 (13)	0.34262 (12)	0.53779 (11)	0.0256 (4)	
N3B	0.00532 (12)	0.24712 (10)	0.22455 (10)	0.0172 (3)	
C1B	−0.05081 (15)	0.33808 (12)	0.36058 (12)	0.0188 (4)	
C2B	0.05373 (15)	0.37806 (13)	0.41839 (12)	0.0194 (4)	
H2B	0.1176	0.3868	0.3932	0.023*	
C3B	0.06095 (15)	0.40445 (13)	0.51366 (12)	0.0194 (4)	
C4B	−0.03079 (15)	0.39308 (13)	0.55503 (12)	0.0204 (4)	
H4B	−0.0242	0.4110	0.6193	0.024*	
C5B	−0.13292 (15)	0.35360 (13)	0.49594 (12)	0.0199 (4)	
C6B	−0.14494 (15)	0.32569 (13)	0.39992 (12)	0.0201 (4)	
H6B	−0.2152	0.2990	0.3624	0.024*	
C7B	−0.06015 (15)	0.31195 (13)	0.25610 (12)	0.0207 (4)	
H7B1	−0.1374	0.2791	0.2262	0.025*	
H7B2	−0.0342	0.3719	0.2380	0.025*	
C8B	−0.05248 (15)	0.14769 (13)	0.22970 (12)	0.0194 (4)	
H8B1	−0.1185	0.1185	0.1813	0.023*	
H8B2	−0.0763	0.1537	0.2890	0.023*	
C9B	0.01553 (15)	0.07838 (13)	0.21962 (12)	0.0216 (4)	
H9B1	−0.0302	0.0132	0.2187	0.026*	
H9B2	0.0419	0.0730	0.1615	0.026*	
C10B	0.02250 (15)	0.24609 (14)	0.12945 (12)	0.0221 (4)	
H10C	−0.0476	0.2371	0.0910	0.026*	
H10D	0.0470	0.1904	0.1035	0.026*	
C11B	0.10692 (15)	0.33980 (14)	0.12651 (13)	0.0239 (4)	
H11C	0.1058	0.3391	0.0621	0.029*	
H11D	0.0850	0.3955	0.1570	0.029*	
O1C	0.71541 (12)	0.14009 (13)	0.03272 (10)	0.0415 (4)	
O2C	0.63713 (11)	0.17727 (10)	0.14992 (9)	0.0291 (3)	
O3C	0.23961 (11)	0.11558 (11)	0.09406 (9)	0.0310 (3)	
O4C	0.14677 (11)	0.06031 (12)	−0.04668 (10)	0.0366 (4)	
O5C	0.59499 (11)	−0.10538 (10)	−0.23968 (9)	0.0235 (3)	
HO5C	0.651 (2)	−0.0576 (19)	−0.2402 (17)	0.050 (8)*	
O6C	0.71965 (11)	0.16752 (10)	−0.31633 (9)	0.0238 (3)	
HO6C	0.742 (2)	0.2218 (19)	−0.2704 (17)	0.049 (7)*	
N1C	0.63519 (13)	0.15101 (12)	0.06620 (11)	0.0243 (3)	
N2C	0.23376 (13)	0.09121 (11)	0.01026 (10)	0.0219 (3)	
N3C	0.50315 (12)	0.04539 (11)	−0.29170 (10)	0.0191 (3)	
C1C	0.43479 (16)	0.10726 (14)	−0.15105 (12)	0.0220 (4)	
C2C	0.53302 (16)	0.12484 (14)	−0.09012 (13)	0.0233 (4)	
H2C	0.5996	0.1328	−0.1113	0.028*	
C3C	0.53039 (15)	0.13036 (13)	0.00218 (12)	0.0200 (4)	
C4C	0.43423 (15)	0.11927 (12)	0.03818 (12)	0.0192 (4)	
H4C	0.4342	0.1240	0.1006	0.023*	
C5C	0.33861 (15)	0.10077 (13)	−0.02452 (12)	0.0187 (4)	
C6C	0.33649 (15)	0.09387 (13)	−0.11784 (12)	0.0211 (4)	
H6C	0.2698	0.0804	−0.1578	0.025*	
C7C	0.43702 (17)	0.10559 (15)	−0.25104 (12)	0.0257 (4)	
H7C1	0.3618	0.0790	−0.2867	0.031*	
H7C2	0.4682	0.1725	−0.2542	0.031*	
C8C	0.44264 (15)	−0.05947 (14)	−0.30705 (13)	0.0240 (4)	
H8C1	0.3839	−0.0816	−0.3614	0.029*	
H8C2	0.4083	−0.0668	−0.2541	0.029*	
C9C	0.51532 (16)	−0.12435 (14)	−0.32084 (13)	0.0249 (4)	
H9C1	0.4690	−0.1929	−0.3388	0.030*	
H9C2	0.5535	−0.1139	−0.3712	0.030*	
C10C	0.52879 (15)	0.06382 (14)	−0.37885 (12)	0.0207 (4)	
H10E	0.4612	0.0610	−0.4170	0.025*	
H10F	0.5542	0.0115	−0.4120	0.025*	
C11C	0.61517 (15)	0.16122 (14)	−0.36682 (13)	0.0242 (4)	
H11E	0.6243	0.1697	−0.4274	0.029*	
H11F	0.5904	0.2141	−0.3339	0.029*	
O1D	0.26689 (11)	0.28080 (10)	−0.05807 (9)	0.0261 (3)	
O2D	0.36713 (12)	0.35765 (11)	−0.14003 (9)	0.0328 (3)	
O3D	0.76729 (11)	0.52132 (9)	−0.00954 (9)	0.0262 (3)	
O4D	0.83655 (11)	0.44537 (11)	0.07628 (10)	0.0302 (3)	
O5D	0.36167 (11)	0.54780 (10)	0.21380 (9)	0.0256 (3)	
HO5D	0.324 (2)	0.4859 (19)	0.2020 (17)	0.046 (7)*	
O6D	0.24153 (11)	0.31012 (10)	0.33530 (9)	0.0229 (3)	
HO6D	0.194 (2)	0.250 (2)	0.3221 (19)	0.062 (9)*	
N1D	0.35569 (13)	0.33096 (11)	−0.06984 (10)	0.0212 (3)	
N2D	0.75843 (13)	0.46424 (11)	0.03815 (10)	0.0215 (3)	
N3D	0.45683 (12)	0.41919 (11)	0.29645 (10)	0.0179 (3)	
C1D	0.53232 (15)	0.36765 (12)	0.15762 (12)	0.0180 (4)	
C2D	0.43934 (15)	0.34551 (12)	0.08910 (12)	0.0185 (4)	
H2D	0.3684	0.3218	0.1006	0.022*	
C3D	0.45435 (15)	0.35935 (12)	0.00347 (12)	0.0185 (4)	
C4D	0.55746 (15)	0.39844 (12)	−0.01705 (12)	0.0194 (4)	
H4D	0.5658	0.4108	−0.0737	0.023*	
C5D	0.64670 (15)	0.41768 (12)	0.05248 (12)	0.0189 (4)	
C6D	0.63704 (15)	0.40213 (12)	0.13824 (12)	0.0188 (4)	
H6D	0.7000	0.4147	0.1823	0.023*	
C7D	0.51909 (15)	0.35664 (13)	0.25278 (12)	0.0191 (4)	
H7D1	0.4802	0.2882	0.2474	0.023*	
H7D2	0.5917	0.3750	0.2916	0.023*	
C8D	0.51305 (15)	0.52482 (13)	0.30997 (13)	0.0221 (4)	
H8D1	0.5571	0.5325	0.2625	0.027*	
H8D2	0.5627	0.5532	0.3700	0.027*	
C9D	0.42915 (17)	0.57882 (15)	0.30486 (14)	0.0304 (5)	
H9D1	0.3819	0.5670	0.3495	0.036*	
H9D2	0.4678	0.6492	0.3216	0.036*	
C10D	0.43487 (15)	0.40129 (14)	0.38478 (12)	0.0236 (4)	
H10G	0.4132	0.4551	0.4201	0.028*	
H10H	0.5029	0.4016	0.4203	0.028*	
C11D	0.34582 (15)	0.30534 (15)	0.37407 (13)	0.0247 (4)	
H11G	0.3626	0.2514	0.3337	0.030*	
H11H	0.3428	0.2934	0.4342	0.030*	

### Atomic displacement parameters (Å^2^)

**Table:**

	*U*^11^	*U*^22^	*U*^33^	*U*^12^	*U*^13^	*U*^23^
O1A	0.0246 (8)	0.0631 (11)	0.0326 (8)	0.0215 (8)	−0.0050 (6)	−0.0022 (7)
O2A	0.0309 (8)	0.0278 (7)	0.0182 (7)	0.0051 (6)	0.0003 (6)	0.0019 (6)
O3A	0.0320 (8)	0.0423 (9)	0.0269 (8)	0.0182 (7)	0.0108 (6)	0.0040 (6)
O4A	0.0181 (7)	0.0400 (8)	0.0367 (8)	0.0072 (6)	0.0055 (6)	0.0078 (7)
O5A	0.0234 (7)	0.0207 (7)	0.0262 (7)	0.0066 (6)	0.0014 (6)	0.0070 (6)
O6A	0.0241 (7)	0.0250 (7)	0.0283 (7)	0.0125 (6)	0.0006 (6)	0.0060 (6)
N1A	0.0230 (9)	0.0280 (9)	0.0199 (8)	0.0073 (7)	−0.0005 (7)	0.0061 (7)
N2A	0.0218 (9)	0.0304 (9)	0.0242 (8)	0.0108 (7)	0.0074 (7)	0.0115 (7)
N3A	0.0187 (8)	0.0179 (7)	0.0173 (7)	0.0077 (6)	0.0034 (6)	0.0039 (6)
C1A	0.0209 (9)	0.0176 (9)	0.0177 (9)	0.0067 (7)	0.0029 (7)	0.0072 (7)
C2A	0.0178 (9)	0.0191 (9)	0.0197 (9)	0.0064 (7)	0.0036 (7)	0.0076 (7)
C3A	0.0186 (9)	0.0193 (9)	0.0188 (9)	0.0043 (7)	−0.0006 (7)	0.0076 (7)
C4A	0.0245 (10)	0.0219 (9)	0.0175 (9)	0.0093 (8)	0.0032 (7)	0.0063 (7)
C5A	0.0197 (9)	0.0227 (9)	0.0212 (9)	0.0084 (8)	0.0076 (7)	0.0093 (7)
C6A	0.0188 (9)	0.0188 (9)	0.0192 (9)	0.0042 (7)	0.0001 (7)	0.0060 (7)
C7A	0.0182 (9)	0.0203 (9)	0.0194 (9)	0.0060 (7)	0.0012 (7)	0.0040 (7)
C8A	0.0217 (9)	0.0208 (9)	0.0181 (9)	0.0095 (8)	0.0016 (7)	0.0048 (7)
C9A	0.0260 (10)	0.0225 (9)	0.0200 (9)	0.0098 (8)	0.0046 (8)	0.0068 (7)
C10A	0.0201 (9)	0.0205 (9)	0.0172 (9)	0.0072 (7)	0.0026 (7)	0.0027 (7)
C11A	0.0239 (10)	0.0212 (9)	0.0216 (9)	0.0098 (8)	0.0054 (8)	0.0049 (7)
O1B	0.0185 (7)	0.0506 (10)	0.0315 (8)	0.0069 (7)	0.0032 (6)	0.0047 (7)
O2B	0.0325 (8)	0.0397 (8)	0.0182 (7)	0.0049 (7)	−0.0028 (6)	0.0071 (6)
O3B	0.0305 (8)	0.0576 (10)	0.0234 (8)	0.0154 (7)	0.0105 (6)	0.0106 (7)
O4B	0.0178 (7)	0.0424 (9)	0.0322 (8)	0.0062 (6)	0.0037 (6)	0.0100 (7)
O5B	0.0226 (7)	0.0239 (7)	0.0218 (7)	0.0102 (6)	0.0001 (5)	0.0033 (5)
O6B	0.0185 (7)	0.0361 (8)	0.0222 (7)	0.0033 (6)	0.0012 (5)	0.0120 (6)
N1B	0.0207 (8)	0.0226 (8)	0.0243 (9)	0.0073 (7)	−0.0006 (7)	0.0034 (6)
N2B	0.0236 (9)	0.0304 (9)	0.0242 (9)	0.0093 (7)	0.0065 (7)	0.0089 (7)
N3B	0.0186 (8)	0.0181 (7)	0.0160 (7)	0.0072 (6)	0.0051 (6)	0.0046 (6)
C1B	0.0218 (9)	0.0153 (8)	0.0201 (9)	0.0091 (7)	0.0024 (7)	0.0034 (7)
C2B	0.0186 (9)	0.0183 (9)	0.0214 (9)	0.0080 (7)	0.0044 (7)	0.0036 (7)
C3B	0.0192 (9)	0.0163 (9)	0.0218 (9)	0.0073 (7)	0.0003 (7)	0.0039 (7)
C4B	0.0247 (10)	0.0177 (9)	0.0192 (9)	0.0084 (8)	0.0031 (7)	0.0049 (7)
C5B	0.0204 (9)	0.0181 (9)	0.0239 (9)	0.0086 (7)	0.0069 (7)	0.0071 (7)
C6B	0.0193 (9)	0.0159 (9)	0.0225 (9)	0.0057 (7)	−0.0004 (7)	0.0028 (7)
C7B	0.0211 (9)	0.0215 (9)	0.0198 (9)	0.0090 (8)	0.0014 (7)	0.0052 (7)
C8B	0.0184 (9)	0.0181 (9)	0.0210 (9)	0.0052 (7)	0.0047 (7)	0.0047 (7)
C9B	0.0215 (10)	0.0213 (9)	0.0198 (9)	0.0073 (8)	0.0006 (7)	0.0030 (7)
C10B	0.0197 (9)	0.0286 (10)	0.0156 (9)	0.0047 (8)	0.0024 (7)	0.0062 (7)
C11B	0.0185 (9)	0.0309 (10)	0.0226 (9)	0.0052 (8)	0.0013 (7)	0.0125 (8)
O1C	0.0233 (8)	0.0742 (12)	0.0319 (8)	0.0229 (8)	0.0080 (6)	0.0145 (8)
O2C	0.0251 (7)	0.0395 (8)	0.0198 (7)	0.0065 (6)	0.0013 (6)	0.0092 (6)
O3C	0.0266 (8)	0.0480 (9)	0.0216 (7)	0.0148 (7)	0.0089 (6)	0.0106 (6)
O4C	0.0175 (7)	0.0495 (9)	0.0298 (8)	0.0033 (7)	0.0001 (6)	−0.0011 (7)
O5C	0.0215 (7)	0.0249 (7)	0.0226 (7)	0.0052 (6)	0.0003 (5)	0.0088 (6)
O6C	0.0211 (7)	0.0240 (7)	0.0253 (7)	0.0088 (6)	0.0005 (6)	0.0053 (6)
N1C	0.0196 (8)	0.0302 (9)	0.0238 (9)	0.0087 (7)	0.0033 (7)	0.0086 (7)
N2C	0.0199 (8)	0.0212 (8)	0.0228 (8)	0.0058 (7)	0.0034 (7)	0.0045 (6)
N3C	0.0223 (8)	0.0209 (8)	0.0159 (7)	0.0098 (6)	0.0046 (6)	0.0045 (6)
C1C	0.0263 (10)	0.0239 (9)	0.0187 (9)	0.0130 (8)	0.0056 (8)	0.0054 (7)
C2C	0.0226 (10)	0.0287 (10)	0.0231 (10)	0.0136 (8)	0.0079 (8)	0.0079 (8)
C3C	0.0193 (9)	0.0212 (9)	0.0196 (9)	0.0086 (8)	0.0015 (7)	0.0046 (7)
C4C	0.0227 (10)	0.0165 (9)	0.0179 (9)	0.0071 (7)	0.0034 (7)	0.0033 (7)
C5C	0.0186 (9)	0.0157 (8)	0.0212 (9)	0.0054 (7)	0.0057 (7)	0.0033 (7)
C6C	0.0224 (10)	0.0206 (9)	0.0190 (9)	0.0099 (8)	−0.0005 (7)	0.0020 (7)
C7C	0.0294 (11)	0.0345 (11)	0.0193 (9)	0.0188 (9)	0.0051 (8)	0.0083 (8)
C8C	0.0190 (10)	0.0257 (10)	0.0245 (10)	0.0044 (8)	−0.0016 (8)	0.0077 (8)
C9C	0.0289 (11)	0.0221 (10)	0.0200 (9)	0.0079 (8)	−0.0042 (8)	0.0037 (7)
C10C	0.0207 (10)	0.0270 (10)	0.0149 (8)	0.0099 (8)	0.0025 (7)	0.0048 (7)
C11C	0.0215 (10)	0.0290 (10)	0.0250 (10)	0.0105 (8)	0.0032 (8)	0.0110 (8)
O1D	0.0178 (7)	0.0279 (7)	0.0306 (7)	0.0060 (6)	0.0012 (6)	0.0080 (6)
O2D	0.0302 (8)	0.0427 (9)	0.0231 (7)	0.0065 (7)	−0.0008 (6)	0.0140 (6)
O3D	0.0295 (8)	0.0229 (7)	0.0270 (7)	0.0059 (6)	0.0104 (6)	0.0091 (6)
O4D	0.0192 (7)	0.0399 (8)	0.0328 (8)	0.0098 (6)	0.0045 (6)	0.0128 (6)
O5D	0.0224 (7)	0.0228 (7)	0.0291 (7)	0.0081 (6)	−0.0033 (6)	0.0054 (6)
O6D	0.0170 (7)	0.0255 (7)	0.0250 (7)	0.0064 (6)	0.0024 (5)	0.0066 (6)
N1D	0.0223 (8)	0.0209 (8)	0.0201 (8)	0.0090 (7)	0.0013 (6)	0.0042 (6)
N2D	0.0207 (8)	0.0222 (8)	0.0194 (8)	0.0054 (7)	0.0045 (6)	0.0035 (6)
N3D	0.0169 (8)	0.0182 (7)	0.0178 (7)	0.0059 (6)	0.0039 (6)	0.0032 (6)
C1D	0.0228 (9)	0.0138 (8)	0.0187 (9)	0.0080 (7)	0.0048 (7)	0.0039 (7)
C2D	0.0167 (9)	0.0154 (8)	0.0235 (9)	0.0054 (7)	0.0055 (7)	0.0047 (7)
C3D	0.0187 (9)	0.0155 (8)	0.0204 (9)	0.0071 (7)	0.0005 (7)	0.0029 (7)
C4D	0.0249 (10)	0.0162 (9)	0.0175 (9)	0.0074 (7)	0.0052 (7)	0.0041 (7)
C5D	0.0186 (9)	0.0152 (8)	0.0218 (9)	0.0047 (7)	0.0051 (7)	0.0035 (7)
C6D	0.0197 (9)	0.0169 (9)	0.0179 (9)	0.0069 (7)	0.0002 (7)	0.0021 (7)
C7D	0.0192 (9)	0.0198 (9)	0.0194 (9)	0.0080 (7)	0.0036 (7)	0.0058 (7)
C8D	0.0199 (9)	0.0189 (9)	0.0245 (10)	0.0067 (7)	−0.0008 (7)	0.0024 (7)
C9D	0.0289 (11)	0.0254 (10)	0.0307 (11)	0.0126 (9)	−0.0066 (9)	−0.0019 (8)
C10D	0.0182 (9)	0.0345 (11)	0.0151 (9)	0.0077 (8)	0.0014 (7)	0.0034 (8)
C11D	0.0205 (10)	0.0372 (11)	0.0229 (10)	0.0129 (8)	0.0063 (8)	0.0152 (8)

### Geometric parameters (Å, °)

**Table:**

O1A—N1A	1.225 (2)	O1C—N1C	1.227 (2)
O2A—N1A	1.2309 (19)	O2C—N1C	1.2257 (19)
O3A—N2A	1.228 (2)	O3C—N2C	1.2177 (19)
O4A—N2A	1.227 (2)	O4C—N2C	1.225 (2)
O5A—C9A	1.436 (2)	O5C—C9C	1.421 (2)
O5A—HO5A	0.89 (3)	O5C—HO5C	0.85 (3)
O6A—C11A	1.436 (2)	O6C—C11C	1.432 (2)
O6A—HO6A	0.84 (3)	O6C—HO6C	0.87 (3)
N1A—C3A	1.469 (2)	N1C—C3C	1.473 (2)
N2A—C5A	1.477 (2)	N2C—C5C	1.478 (2)
N3A—C7A	1.465 (2)	N3C—C10C	1.463 (2)
N3A—C8A	1.471 (2)	N3C—C7C	1.468 (2)
N3A—C10A	1.479 (2)	N3C—C8C	1.469 (2)
C1A—C2A	1.394 (2)	C1C—C6C	1.387 (3)
C1A—C6A	1.398 (2)	C1C—C2C	1.393 (3)
C1A—C7A	1.513 (2)	C1C—C7C	1.510 (2)
C2A—C3A	1.383 (2)	C2C—C3C	1.383 (3)
C2A—H2A	0.9300	C2C—H2C	0.9300
C3A—C4A	1.383 (3)	C3C—C4C	1.385 (2)
C4A—C5A	1.379 (3)	C4C—C5C	1.379 (2)
C4A—H4A	0.9300	C4C—H4C	0.9300
C5A—C6A	1.386 (2)	C5C—C6C	1.387 (2)
C6A—H6A	0.9300	C6C—H6C	0.9300
C7A—H7A1	0.9700	C7C—H7C1	0.9700
C7A—H7A2	0.9700	C7C—H7C2	0.9700
C8A—C9A	1.514 (3)	C8C—C9C	1.507 (3)
C8A—H8A1	0.9700	C8C—H8C1	0.9700
C8A—H8A2	0.9700	C8C—H8C2	0.9700
C9A—H9A1	0.9700	C9C—H9C1	0.9700
C9A—H9A2	0.9700	C9C—H9C2	0.9700
C10A—C11A	1.508 (2)	C10C—C11C	1.501 (3)
C10A—H10A	0.9700	C10C—H10E	0.9700
C10A—H10B	0.9700	C10C—H10F	0.9700
C11A—H11A	0.9700	C11C—H11E	0.9700
C11A—H11B	0.9700	C11C—H11F	0.9700
O1B—N1B	1.224 (2)	O1D—N1D	1.2279 (19)
O2B—N1B	1.228 (2)	O2D—N1D	1.2290 (19)
O3B—N2B	1.226 (2)	O3D—N2D	1.2262 (19)
O4B—N2B	1.223 (2)	O4D—N2D	1.232 (2)
O5B—C9B	1.435 (2)	O5D—C9D	1.435 (2)
O5B—HO5B	0.84 (3)	O5D—HO5D	0.87 (3)
O6B—C11B	1.418 (2)	O6D—C11D	1.425 (2)
O6B—HO6B	0.87 (3)	O6D—HO6D	0.88 (3)
N1B—C3B	1.473 (2)	N1D—C3D	1.472 (2)
N2B—C5B	1.473 (2)	N2D—C5D	1.468 (2)
N3B—C7B	1.470 (2)	N3D—C10D	1.466 (2)
N3B—C8B	1.471 (2)	N3D—C7D	1.468 (2)
N3B—C10B	1.477 (2)	N3D—C8D	1.473 (2)
C1B—C6B	1.389 (3)	C1D—C6D	1.385 (2)
C1B—C2B	1.396 (2)	C1D—C2D	1.393 (2)
C1B—C7B	1.515 (2)	C1D—C7D	1.510 (2)
C2B—C3B	1.383 (2)	C2D—C3D	1.385 (2)
C2B—H2B	0.9300	C2D—H2D	0.9300
C3B—C4B	1.380 (3)	C3D—C4D	1.386 (3)
C4B—C5B	1.381 (3)	C4D—C5D	1.377 (3)
C4B—H4B	0.9300	C4D—H4D	0.9300
C5B—C6B	1.388 (2)	C5D—C6D	1.384 (2)
C6B—H6B	0.9300	C6D—H6D	0.9300
C7B—H7B1	0.9700	C7D—H7D1	0.9700
C7B—H7B2	0.9700	C7D—H7D2	0.9700
C8B—C9B	1.510 (2)	C8D—C9D	1.512 (3)
C8B—H8B1	0.9700	C8D—H8D1	0.9700
C8B—H8B2	0.9700	C8D—H8D2	0.9700
C9B—H9B1	0.9700	C9D—H9D1	0.9700
C9B—H9B2	0.9700	C9D—H9D2	0.9700
C10B—C11B	1.510 (3)	C10D—C11D	1.506 (3)
C10B—H10C	0.9700	C10D—H10G	0.9700
C10B—H10D	0.9700	C10D—H10H	0.9700
C11B—H11C	0.9700	C11D—H11G	0.9700
C11B—H11D	0.9700	C11D—H11H	0.9700
			
C9A—O5A—HO5A	106.4 (16)	C9C—O5C—HO5C	106.9 (17)
C11A—O6A—HO6A	108.6 (18)	C11C—O6C—HO6C	107.9 (17)
O1A—N1A—O2A	124.27 (16)	O2C—N1C—O1C	123.68 (16)
O1A—N1A—C3A	117.37 (15)	O2C—N1C—C3C	118.17 (15)
O2A—N1A—C3A	118.36 (15)	O1C—N1C—C3C	118.14 (15)
O4A—N2A—O3A	124.24 (16)	O3C—N2C—O4C	124.11 (16)
O4A—N2A—C5A	117.86 (15)	O3C—N2C—C5C	117.69 (15)
O3A—N2A—C5A	117.90 (15)	O4C—N2C—C5C	118.20 (15)
C7A—N3A—C8A	110.49 (14)	C10C—N3C—C7C	110.89 (14)
C7A—N3A—C10A	109.39 (13)	C10C—N3C—C8C	110.55 (14)
C8A—N3A—C10A	111.12 (13)	C7C—N3C—C8C	109.54 (15)
C2A—C1A—C6A	119.19 (16)	C6C—C1C—C2C	119.03 (17)
C2A—C1A—C7A	120.14 (16)	C6C—C1C—C7C	121.06 (17)
C6A—C1A—C7A	120.60 (16)	C2C—C1C—C7C	119.89 (17)
C3A—C2A—C1A	118.93 (16)	C3C—C2C—C1C	119.23 (17)
C3A—C2A—H2A	120.5	C3C—C2C—H2C	120.4
C1A—C2A—H2A	120.5	C1C—C2C—H2C	120.4
C2A—C3A—C4A	123.74 (17)	C2C—C3C—C4C	123.33 (17)
C2A—C3A—N1A	118.26 (16)	C2C—C3C—N1C	118.64 (16)
C4A—C3A—N1A	118.00 (16)	C4C—C3C—N1C	118.01 (16)
C5A—C4A—C3A	115.60 (16)	C5C—C4C—C3C	115.68 (16)
C5A—C4A—H4A	122.2	C5C—C4C—H4C	122.2
C3A—C4A—H4A	122.2	C3C—C4C—H4C	122.2
C4A—C5A—C6A	123.57 (17)	C4C—C5C—C6C	123.32 (17)
C4A—C5A—N2A	117.79 (16)	C4C—C5C—N2C	118.22 (15)
C6A—C5A—N2A	118.62 (16)	C6C—C5C—N2C	118.42 (16)
C5A—C6A—C1A	118.95 (16)	C5C—C6C—C1C	119.38 (17)
C5A—C6A—H6A	120.5	C5C—C6C—H6C	120.3
C1A—C6A—H6A	120.5	C1C—C6C—H6C	120.3
N3A—C7A—C1A	112.46 (14)	N3C—C7C—C1C	111.20 (15)
N3A—C7A—H7A1	109.1	N3C—C7C—H7C1	109.4
C1A—C7A—H7A1	109.1	C1C—C7C—H7C1	109.4
N3A—C7A—H7A2	109.1	N3C—C7C—H7C2	109.4
C1A—C7A—H7A2	109.1	C1C—C7C—H7C2	109.4
H7A1—C7A—H7A2	107.8	H7C1—C7C—H7C2	108.0
N3A—C8A—C9A	112.54 (15)	N3C—C8C—C9C	113.34 (15)
N3A—C8A—H8A1	109.1	N3C—C8C—H8C1	108.9
C9A—C8A—H8A1	109.1	C9C—C8C—H8C1	108.9
N3A—C8A—H8A2	109.1	N3C—C8C—H8C2	108.9
C9A—C8A—H8A2	109.1	C9C—C8C—H8C2	108.9
H8A1—C8A—H8A2	107.8	H8C1—C8C—H8C2	107.7
O5A—C9A—C8A	112.84 (14)	O5C—C9C—C8C	112.65 (15)
O5A—C9A—H9A1	109.0	O5C—C9C—H9C1	109.1
C8A—C9A—H9A1	109.0	C8C—C9C—H9C1	109.1
O5A—C9A—H9A2	109.0	O5C—C9C—H9C2	109.1
C8A—C9A—H9A2	109.0	C8C—C9C—H9C2	109.1
H9A1—C9A—H9A2	107.8	H9C1—C9C—H9C2	107.8
N3A—C10A—C11A	112.37 (14)	N3C—C10C—C11C	114.25 (15)
N3A—C10A—H10A	109.1	N3C—C10C—H10E	108.7
C11A—C10A—H10A	109.1	C11C—C10C—H10E	108.7
N3A—C10A—H10B	109.1	N3C—C10C—H10F	108.7
C11A—C10A—H10B	109.1	C11C—C10C—H10F	108.7
H10A—C10A—H10B	107.9	H10E—C10C—H10F	107.6
O6A—C11A—C10A	108.68 (15)	O6C—C11C—C10C	111.55 (15)
O6A—C11A—H11A	110.0	O6C—C11C—H11E	109.3
C10A—C11A—H11A	110.0	C10C—C11C—H11E	109.3
O6A—C11A—H11B	110.0	O6C—C11C—H11F	109.3
C10A—C11A—H11B	110.0	C10C—C11C—H11F	109.3
H11A—C11A—H11B	108.3	H11E—C11C—H11F	108.0
C9B—O5B—HO5B	108.4 (17)	C9D—O5D—HO5D	107.7 (16)
C11B—O6B—HO6B	109.5 (18)	C11D—O6D—HO6D	105.9 (18)
O1B—N1B—O2B	124.05 (16)	O1D—N1D—O2D	123.66 (15)
O1B—N1B—C3B	118.00 (15)	O1D—N1D—C3D	118.23 (15)
O2B—N1B—C3B	117.93 (15)	O2D—N1D—C3D	118.10 (15)
O4B—N2B—O3B	124.49 (16)	O3D—N2D—O4D	124.70 (16)
O4B—N2B—C5B	118.26 (15)	O3D—N2D—C5D	117.08 (15)
O3B—N2B—C5B	117.25 (15)	O4D—N2D—C5D	118.20 (15)
C7B—N3B—C8B	108.67 (13)	C10D—N3D—C7D	110.61 (14)
C7B—N3B—C10B	110.39 (14)	C10D—N3D—C8D	110.60 (14)
C8B—N3B—C10B	111.62 (13)	C7D—N3D—C8D	112.55 (14)
C6B—C1B—C2B	119.34 (16)	C6D—C1D—C2D	119.29 (16)
C6B—C1B—C7B	120.91 (16)	C6D—C1D—C7D	120.34 (16)
C2B—C1B—C7B	119.72 (16)	C2D—C1D—C7D	120.35 (16)
C3B—C2B—C1B	119.07 (17)	C3D—C2D—C1D	118.90 (16)
C3B—C2B—H2B	120.5	C3D—C2D—H2D	120.5
C1B—C2B—H2B	120.5	C1D—C2D—H2D	120.5
C4B—C3B—C2B	123.10 (17)	C2D—C3D—C4D	123.53 (17)
C4B—C3B—N1B	117.71 (16)	C2D—C3D—N1D	118.36 (16)
C2B—C3B—N1B	119.16 (16)	C4D—C3D—N1D	118.12 (16)
C3B—C4B—C5B	116.40 (16)	C5D—C4D—C3D	115.22 (16)
C3B—C4B—H4B	121.8	C5D—C4D—H4D	122.4
C5B—C4B—H4B	121.8	C3D—C4D—H4D	122.4
C4B—C5B—C6B	122.89 (17)	C4D—C5D—C6D	123.80 (17)
C4B—C5B—N2B	117.80 (16)	C4D—C5D—N2D	118.53 (16)
C6B—C5B—N2B	119.30 (16)	C6D—C5D—N2D	117.50 (16)
C5B—C6B—C1B	119.20 (17)	C5D—C6D—C1D	119.13 (17)
C5B—C6B—H6B	120.4	C5D—C6D—H6D	120.4
C1B—C6B—H6B	120.4	C1D—C6D—H6D	120.4
N3B—C7B—C1B	111.27 (14)	N3D—C7D—C1D	110.74 (14)
N3B—C7B—H7B1	109.4	N3D—C7D—H7D1	109.5
C1B—C7B—H7B1	109.4	C1D—C7D—H7D1	109.5
N3B—C7B—H7B2	109.4	N3D—C7D—H7D2	109.5
C1B—C7B—H7B2	109.4	C1D—C7D—H7D2	109.5
H7B1—C7B—H7B2	108.0	H7D1—C7D—H7D2	108.1
N3B—C8B—C9B	114.87 (15)	N3D—C8D—C9D	110.54 (15)
N3B—C8B—H8B1	108.6	N3D—C8D—H8D1	109.5
C9B—C8B—H8B1	108.5	C9D—C8D—H8D1	109.5
N3B—C8B—H8B2	108.5	N3D—C8D—H8D2	109.5
C9B—C8B—H8B2	108.5	C9D—C8D—H8D2	109.5
H8B1—C8B—H8B2	107.5	H8D1—C8D—H8D2	108.1
O5B—C9B—C8B	109.72 (14)	O5D—C9D—C8D	112.01 (15)
O5B—C9B—H9B1	109.7	O5D—C9D—H9D1	109.2
C8B—C9B—H9B1	109.7	C8D—C9D—H9D1	109.2
O5B—C9B—H9B2	109.7	O5D—C9D—H9D2	109.2
C8B—C9B—H9B2	109.7	C8D—C9D—H9D2	109.2
H9B1—C9B—H9B2	108.2	H9D1—C9D—H9D2	107.9
N3B—C10B—C11B	112.66 (15)	N3D—C10D—C11D	113.96 (15)
N3B—C10B—H10C	109.1	N3D—C10D—H10G	108.8
C11B—C10B—H10C	109.1	C11D—C10D—H10G	108.8
N3B—C10B—H10D	109.1	N3D—C10D—H10H	108.8
C11B—C10B—H10D	109.1	C11D—C10D—H10H	108.8
H10C—C10B—H10D	107.8	H10G—C10D—H10H	107.7
O6B—C11B—C10B	114.53 (15)	O6D—C11D—C10D	109.34 (15)
O6B—C11B—H11C	108.6	O6D—C11D—H11G	109.8
C10B—C11B—H11C	108.6	C10D—C11D—H11G	109.8
O6B—C11B—H11D	108.6	O6D—C11D—H11H	109.8
C10B—C11B—H11D	108.6	C10D—C11D—H11H	109.8
H11C—C11B—H11D	107.6	H11G—C11D—H11H	108.3

### Hydrogen-bond geometry (Å, °)

**Table:**

*D*—H···*A*	*D*—H	H···*A*	*D*···*A*	*D*—H···*A*
O5A—HO5A···O6C^i^	0.89 (3)	1.85 (3)	2.7343 (19)	174 (2)
O6A—HO6A···O5D	0.84 (3)	1.93 (3)	2.7575 (19)	174 (3)
O5B—HO5B···O5A^ii^	0.84 (3)	2.07 (3)	2.8892 (19)	165 (2)
O6B—HO6B···O6D	0.87 (3)	1.89 (3)	2.7529 (19)	170 (3)
O5C—HO5C···O5A^i^	0.85 (3)	2.02 (3)	2.8643 (19)	171 (2)
O6C—HO6C···O6A^i^	0.87 (3)	1.89 (3)	2.7553 (19)	170 (2)
O5D—HO5D···O6B	0.87 (3)	1.94 (3)	2.802 (2)	170 (2)
O6D—HO6D···O5B	0.88 (3)	1.90 (3)	2.7804 (19)	173 (3)

Symmetry codes: (i) −*x*+1, −*y*+1, −*z*; (ii) *x*, *y*−1, *z*.
